# Seven mistakes and potential solutions in epidemiology, including a call for a World Council of Epidemiology and Causality

**DOI:** 10.1186/1742-7622-6-6

**Published:** 2009-12-09

**Authors:** Raj Bhopal

**Affiliations:** 1 Public Health Sciences, Centre for Population Health Sciences, University of Edinburgh, Edinburgh, EH9 8AG, UK

## Abstract

All sciences make mistakes, and epidemiology is no exception. I have chosen 7 illustrative mistakes and derived 7 solutions to avoid them. The mistakes (Roman numerals denoting solutions) are:

1. Failing to provide the context and definitions of study populations. (I Describe the study population in detail)

2. Insufficient attention to evaluation of error. (II Don't pretend error does not exist.)

3. Not demonstrating comparisons are like-for-like. (III Start with detailed comparisons of groups.)

4. Either overstatement or understatement of the case for causality. (IV Never say this design cannot contribute to causality or imply causality is ensured by your design.)

5. Not providing both absolute and relative summary measures. (V Give numbers, rates and comparative measures, and adjust summary measures such as odds ratios appropriately.)

6. In intervention studies not demonstrating general health benefits. (VI Ensure general benefits (mortality/morbidity) before recommending application of cause-specific findings.)

7. Failure to utilise study data to benefit populations. (VII Establish a World Council on Epidemiology to help infer causality from associations and apply the work internationally.)

Analysis of these and other common mistakes is needed to benefit from the increasing discovery of associations that will be multiplying as data mining, linkage, and large-scale scale epidemiology become commonplace.

## Introduction: epidemiological mistakes and solutions

All sciences and scientists make mistakes, and epidemiology and epidemiologists (including this writer) are no exception. Epidemiological mistakes may maim and kill, and sometimes the toll can be massive. The contemporary exemplar of this is hormone replacement therapy (HRT), used by millions of women in the hope of reducing cancer and heart disease [1,2]. Fortunately, the saving of life and health benefits arising from epidemiology, despite its mistakes, seem to outweigh the harm. The lives saved from epidemiological studies of tobacco, for example, possibly outweigh all our mistakes, and the information will save even more lives as tobacco control spreads globally, particularly in Asia[3]. This judgement, however, needs and deserves quantitative evaluation.

Mistakes in epidemiology are mostly simple, but they are often challenging, though only occasionally impossible, to avoid. Every study presents a dilemma on the balance between efficiency and accuracy. More generally, we have the puzzle of why mistakes continue to occur despite much guidance. Mistakes are rarely deliberate, and usually avoidable. I have chosen seven mistakes to illustrate how epidemiology goes wrong, and seven solutions are derived (these numbers were chosen to match the title of the presentation on which this paper is based - seven sins and seven commandments of epidemiology - which chimes with our wider culture on right and wrong). My chosen seven mistakes are a sample on which I have been reflecting [4], and against which I have personally battled, but there is scope for several papers of this kind. Phillipe Grandjean's paper on the seven deadly sins of environmental epidemiology covered human frailties, [5] so this one focuses on the scientific discipline, although the two perspectives overlap. Grandjean constructed his paper around the original sins (gluttony, sloth etc.) as shown in Table 1. The seven mistakes I have chosen illustrate some of the dilemmas facing all of us, and they focus us on principles. These mistakes call for better education and training. I have chosen one or two examples for illustration.

**Table 1:** Common vices in environmental epidemiology

Vice	
Pride	Preoccupation with methodology
Envy	Failure to recognize achievements by others
Wrath	Self-righteous intimidation of competitors
Lust	Desire for academic honors
Gluttony	Excessive craving for publications
Greed	Benefit from vested interests
Sloth	Callousness to injustice

*Extract from Grandjean, P Epidemiology*, Volume 19, Number 1, January 2008 (reference 4)

## Analysis

### Mistake 1: Failing to provide the context and definitions of study populations

Textbooks tell us that epidemiology is a population science, though few discuss why [6], leading me to write a full chapter on the topic in my own textbook [6,7]. Populations differ by place, their characteristics and time. The results may not generalise easily between populations, within subgroups of the same population, or within the same population at different times. This applies particularly to the burden of disease and risk factors, but also to causal understanding. Thus, understanding the kind of population studied is essential. There is nothing more elementary in epidemiology but, nonetheless, this is possibly the commonest of the seven mistakes chosen for this paper.

The solution is:

*(I) State the location and timing of fieldwork and describe the study population in detail, especially age, sex, socio-economic status and ethnic composition*.

Rarely, the study location might be disguised to hide the identity of study participants, especially for rare, stigmatising conditions. If so, authors should use their discretion on the level of detail and, exceptionally, even hide the location. Authors need to justify the decision and inform the reader of why they took it. Otherwise, contextual detail must be given. Timing of fieldwork is essential for examining time trends as the date of publication is a sorry substitute. It is not enough to say the study was done in the USA or even New York, except when the work was indeed national and/or city-wide, respectively. Authors should be specific about the district of the city. Without the study population's details it is not possible to draw appropriate conclusions. This is obvious in relation to applied epidemiology used for health service/public health purposes. It also applies to causal research, where understanding the biological processes, presence of co-risk factors and competing risks of disease and death are so important to generalisation. An association may vary in its strength in different populations, reflecting the presence or absence of co-factors.

#### Examples

When answering specific questions, it is surprising how sparse the scientific literature is, and this applies particularly in low income countries as well as in relation to minority populations within the high income countries. As shown in table 2, in recent reviews my colleagues and I have found that information on time of fieldwork was present in every North American and European cardiovascular cohort study we examined, [8] but missing in a high proportion of studies on minorities and in West Africa [9-12]. By contrast, the cardiovascular cohort studies, particularly those in Europe, were lacking in information on the ethnic composition of the samples. Absence of such basics, especially as investigators move posts and are not always easy to contact, can undermine interpretation of individual publications and weaken both traditional and systematic reviews.

**Table 2:** Missing fieldwork dates and absence of information on ethnicity

**A. Fieldwork dates missing in recent reviews**		
CV cohorts[8]	0/72	0%
		
BP in UK EM groups -- S. Asians [9]	5/12	42%
BP African [10]	8/14	57%
Trends in obesity in W. African [11]	8/28	29%
Trends in diabetes in W. Africa [12]	7/21	33%
**B. Ethnic composition of sample missing**		
No description or discussion of study in relation to ethnicity or race		
CV cohorts [8]	39/72	54%
		
- USA	6/31	19%
- Europe	33/39	80%

Abbreviations:

CV cardiovascular

BP blood pressure

EM ethnic minority

### Mistake 2: Insufficient attention to evaluation of error

Measurement is always imperfect in the empirical sciences, and especially in humans. The most fundamental error is mismeasurement, which is ubiquitous and often unavoidable. We have motivations to ignore error. We want a rapid, inexpensive and conclusive outcome. Seeking out and rectifying errors requires scarce time and resources, and funding (for pilot studies, for example) is not easy to obtain. We want (and need) publication. While acknowledging errors and limitations is good scholarship, openness can lead to rejection of manuscripts during review, particularly if errors could, in retrospect, have been avoided. Where publication is associated with direct financial rewards and prestige, the temptation to gloss over errors is strong. The solution arising is, again, a basic one, but one that needs reinforcement, for it is widely ignored.

Solution II: Don't act as if measurement error does not exist. If possible quantify it. If not, identify that as a limitation of the work. (Remember that lack of cross-cultural validity of measures may lead to serious errors.)

#### Examples

Our potential actions are to ignore errors, acknowledge them but do nothing, make qualitative adjustments in the interpretation - probably through qualifying words such as 'may', 'could', etc. (this is perhaps the commonest response) - or make quantitative adjustments to summary measures. Maldonado argues that making no quantitative adjustment equates to the assumption that the study's imperfections have no important impact on study results, which is mostly untrue. He demonstrates how to quantitatively adjust relative risk using error terms [13]. Such adjustments increase the uncertainty of the summary measures (widening confidence intervals). This makes conclusions more tentative, which is good science but potentially an obstacle to publication and implementation.

The need for cross-cultural validity of self report data is self evident but often ignored [14]. It is less obvious, and more controversial, that the interpretation of physical measures such as BMI, waist, birth weight, and fetal and childhood growth may differ across different populations, particularly by ethnic group [15]. The differential performance of clinical measures, such as the electrocardiogram, is reasonably well established, but has not led to any substantial programme of research to improve matters [16]. The cross-cultural, cross-national validity of biochemical measures, for example, the biological effect of a particular level of cholesterol, and normal values of glucose during the oral glucose tolerance test, has hardly been discussed.

Implementing this solution is a major challenge. The mistake is to ignore the problem.

### Mistake 3: Not demonstrating comparisons are like-for-like, and the problem of confounding

Epidemiologists are fortunate that they study humans. This privilege comes at a price: the (relatively) easy route to causal knowledge through experimentation is largely barred for ethical reasons; the scientific principle of splitting a tissue specimen or a sample of cloned/inbred animals into a control or study group, permitting rigorous like-for-like comparisons, is rarely possible.

The closest practical alternatives in human epidemiology are randomised controlled trials, but even these have error. Even if like-for-like comparison is achieved by randomisation and blinding (both are needed), many factors including the selection of populations, may preclude causal inference to the target population, i.e. external validity. Only a small fraction of epidemiological questions are answerable using trials, either for ethical or resource- and time-related reasons. If comparing like-with-like is not possible, confounding is inevitable. The epidemiological mistake, however, is neither confounding nor failing to control for it - these are not fully in our hands. The mistake is not demonstrating whether comparisons are unlikely or likely to be like-for-like. Like mistakes 1 and 2 this one is also fundamental, and the solution is clear cut.

*Solution III: Prepare a detailed, prior specification of the data to be collected in order to demonstrate the similarities and differences of comparison populations. Start the analysis with such detailed comparisons*.

The paradox is that the first part of this solution is nearly always applied in trials (usually table 1), where it is not essential if the randomisation has worked, but is often missed out in other study designs, where it is imperative for data interpretation. The analysis must not proceed straight to comparisons of risk factor-disease outcome relationships. The strategy of controlling confounding within the (usually) multivariable analysis is not sufficient. The reason for this is that the variables entered are not comprehensive enough and that they are imprecisely measured and categorised. While the idea of residual confounding is known it is not taken seriously enough [17]. How often do authors conclude that the confounders cannot be controlled and that they have refrained from a multivariable analysis because causal conclusions cannot be drawn? That is a rare conclusion, but it should be a common one.

#### Example: alcohol

Behaviours are linked to other behaviours, social circumstances, age, sex and, of course, diseases. We might therefore assume that control of confounding is nigh impossible when we are looking for associations between single behaviours and disease outcomes. Instead of that, we find ourselves in almost endless controversies over repeated studies demonstrating a variety of associations that, in the paraphrased words of the late Petr Skrabanek, can be likened to punching a pillow, whereby the dimple so formed disappears, at which point we then punch it again, and again [18]. An example is the association between alcohol and cardiovascular diseases (CVD), which has been studied intensively for decades. Thousands of papers have resulted, many of them debating the evidence, which remains controversial. The study by Naimi *et al*. of the potential confounding factors using the 2003 Behavioural Risk Factor Surveillance System is illustrative [19]. This large telephone survey permitted examination of moderate drinkers (men 2 drinks, and women 1 drink, per day) and non-drinkers. The study showed that 27 out of 30 CVD-associated factors were significantly more prevalent in non-drinkers. Given imprecision in measurement of confounders, and the many other potential confounders not included in a telephone survey, is it not impossible to adjust for confounders in this and similar contexts? We need to confront such harsh realities. Implementing this solution will be a major advance in interpreting associations.

### Mistake 4: Either overstatement or understatement of the case for causality based on associations

Overstatement of the case for causality is perennial, much discussed but not resolved. The problem is that causal reasoning is not suitable for an algorithm based approach. Counter-factual reasoning clarifies the underpinnings of causal reasoning in epidemiology, but it is theoretical [20-23]. The closest we come to the counterfactual ideal is trials, which are often not possible for ethical, financial or time-related reasons. Whenever possible, we should test out causal hypotheses using trials, but unless we envisage an age of unethical research, based on human experimentation, we will need to improve our conceptual frameworks for causal reasoning, particularly for non-trial epidemiology [20-23]. Causal frameworks in epidemiology, often founded on those of other disciplines, [4] have served us well and deserve to be improved through research rather than being subjected to the destabilising effects of attack and counter-attack. The interpretation of associations needs to be done with great rigour. In relation to mistake 7, and in my conclusion, I recommend that we set up a World Council in Epidemiology and Causality that compiles and evaluates evidence on associations.

Recently, a new problem has emerged: the understatement of the case for causality. Typically, the authors declare that a finding is not causal because the data come from a study of population statistics, or of cross-sectional, case-control or even cohort design. This arises from the too simplistic equating of study design with causal reasoning. As soon as epidemiologists postulate an association, never mind demonstrate that one exists, they are somewhere on the pathway of causal analysis. The key question is where we are on this path, surely the toughest one in epidemiology, and not one that can be evaded. It is made even tougher when trials and observational studies contradict each other. There are many good reasons why this contradiction happens, including the problem of confounding, insufficient study size (power) to demonstrate effects, the possibility that trials have not provided the duration and amount of exposure to the factor of interest to lead to an effect, the possibility that the exposure needs to be at a critical period of life that cannot be mimicked in a trial (for example, the first year), and, of course, that trials may be done in different kinds of populations to those studied in observational studies. The problem of causality is tough and deserves 7 solutions on its own, but I have chosen one.

*Solution IV: Never say that a particular study design cannot contribute to causality or imply that causality is ensured by your design, but provide a judgement based on a theoretical perspective on causality and the world's empirical and theoretical literature*.

Causal understanding may hold firm on the flimsiest of evidence (or even none that we recognise as epidemiology) and be demolished when it derives from large empirical data sets with strong designs (for example, cohorts), as in the example of HRT above [1,2]. A judgement that arises from the world's empirical and theoretical literature demands a discipline to avoid selective reading and rapid production of 'knowledge'. It demands a return to a chronological and thorough review as in a PhD, at least where causality is relevant. Ideas around causation are likely to be in commentaries and reviews, reports and theses, and introductory and discussion sections of empirical writings (not in the data in the results section). Such traditional reviews are surely more suited to in-depth causal thinking, while systematic reviews are better suited to quantifying effects, which is no more than the first step in the long path to declaring an association as causal.

#### Examples

In 1971 Herbst *et al *reported that their case-control study of adenocarcinoma of the vagina in young women showed an association with maternal use of stilbestrol [24]. The case group consisted of 8 girls born in New England hospitals between 1946 - 1951 and treated between 1966 - 1969. They selected 4 controls per case. The data on oestrogens given to mothers in the relevant pregnancy were as follows:

Cases: 7/8

Controls: 0/32

P < 0.00001 (chi squared)

Herbst *et al*'s discussion of their findings was integrated in terms of epidemiology, biology and clinical medicine. Their understated but clear-cut conclusion was that "... the results of this study suggest it is unwise to administer stilbestrol to women early in pregnancy." They did not flinch from the challenge because the case group was so small and the power of the study was low. Equally, they did not hedge their bets by saying case-control studies do not produce causal evidence. Let us contrast this with a recent case-control study.

Ismail *et al *studied risk factors for myocardial infarction (MI) in Pakistan in a case-control study [25]. They highlighted 8 risk factors in the abstract. Of these, six had odds ratios (ORs) = 3 and one had an OR = 0.04 (95% CI: 0.01 - 0.35). They concluded:

"...While this study does not establish a cause-effect relation ... it raises the possibility that several of the associated factors may be modifiable risk factors ...". "Based on our findings, we suggest that stringent ...". They then listed six public health actions based on their risk factors. This claim that the study design does not permit a causal interpretation, while proceeding to interpret it as causal, is not good epidemiology. This kind of contradictory reasoning is common, as shown in the abstracts of the Society for Epidemiological Research (SER) Conference 2008; examples include abstract 77 and abstract 378 [26].

Each risk factor needs to be interpreted using an appropriate causal framework, and with reference to the full scientific literature and not just the study's data. Implementing this solution will be tough, as it requires deep scholarship that transcends the disciplines that underpin causal understanding, including biology, pathology, epidemiology, statistics, social science and philosophy. Authors may be pressured by editors, referees and their own institutions into these mistakes on causality, but they remain responsible for them, so need to resist external pressures.

### Mistake 5: Not providing appropriate, and appropriately adjusted, absolute and relative measures

Accurate age- and sex-specific rates underpin virtually everything we value highly in epidemiology. Other data summaries distort the basic epidemiological reality of such rates. How much time do we spend looking at age- and sex-specific rates, the building blocks of epidemiology? How much do we reflect on the distortions of other forms of data presentation? By choosing one pathway to analysis - usually relative measures such as the relative risk and the odds ratio - we close off other options. As a minimum, however, in recognition of their different messages, we should give absolute and relative measures. Relative measures are particularly prone to distortion, and some, such as the odds ratio, have an inbuilt exaggeration of the association. When we use measures such as ORs we should ensure they are adjusted to give valid relative measures [7,27].

The solution is important for epidemiology:

*Solution V. Give numbers, rates and comparative measures - rates hold primacy - and adjust the summary measure if appropriate*.

#### Examples

I have emphasised the importance of absolute measures, especially for health needs assessment, for more than 20 years [28]. The data in table 3, showing how different priorities seem when examined from an absolute compared to relative risk perspective, continue to surprise both practitioners and researchers [28-30]. The topic remains controversial, especially in the health inequalities arena [31,32].

**Table 3:** Deaths and SMRs* in male immigrants from the indian sub continent (aged 20 and over; total deaths = 4,352)

By rank order of number of deaths (absolute risk approach)			By rank order of SMR (relative risk approach)		
**Cause**	**Number of Deaths** **(SMR)**	**% of Total deaths**	**Cause**	**SMR (Number of Deaths)**	**% of Total deaths**
Ischaemic heart disease	1533 (115)	35.2	Homicide	341 (21)	0.5
Cerebrovascular disease	438 (108)	10.1	Liver and intrahepatic bile duct neoplasm	338 (19)	0.4
Bronchitis, emphysema and asthma	223 (77)	5.1	Tuberculosis	315 (64)	1.5
Neoplasm of the trachea, bronchus and lung	218 (53)	5.0	Diabetes mellitus	188 (55)	1.3
Other non-viral pneumonia	214 (100)	4.9	Neoplasm of buccal cavity and pharynx	178 (28)	0.6
TOTAL	2626	60.3		187	4.3

*Standardised mortality ratios, comparing with the male population of England and Wales, which was by definition 100.

This table is adapted from the version published by Senior and Bhopal (1994) and republished by Bhopal in reference 5.

The odds ratio is particularly prone to giving results that are interpreted very differently (and often wrongly) from the corresponding absolute measure and even the corresponding relative measure. One of many examples that has drawn critical comment, diverting from the main message of the paper, is the work of Barnes and Bero, reporting that there was a strong association between conclusions of review articles and authors' affiliations with the tobacco industry [33]. The published odds ratio was 88.4 (95% CI: 16.4 -476.5). The actual data were that 10 of 75 (13.3%) reviews by non-tobacco-affiliated authors concluded that passive smoking was not harmful to health. By contrast 29 of 31 (96.1%) reviews by tobacco-affiliated authors reached this conclusion. The result from the odds ratio is at odds with actual data, the prevalence ratio (7.3), and the absolute risk difference (82.8%). This odds ratio was misinterpreted in the BMJ as a risk ratio.

The question of what odds ratios measure in case-control studies has been reviewed recently, with the conclusion that there is insufficient attention to this issue [34]. My work with Katherine MacGilchrist and Robin Prescott has shown that about 50% of the reports of odds ratios in four major medical journals in the year 2000 were contrary to epidemiological guidance on their presentation and/or interpretation (unpublished).

### Mistake 6: Making public health recommendations from intervention studies that show specific benefits but not do not demonstrate general health benefits

We live in a specialist's world. We have cardiologists and cardiovascular epidemiologists who want to control cardiovascular disorders. But there is no point if there is no net benefit, for example, if costs and side-effects balance or even outweigh the benefits. We really need, at least from a health and health care perspective, *a life expectancy and health expectancy specialism *to dominate the scene. Conclusions from research on specific outcomes draw important but still-limited conclusions, and these should be tested against the goal of general benefits. Of course, specific outcomes help in the causal endeavour, where contradictory results are particularly informative, raising questions about why a particular factor increases one disease or outcome, while decreasing another. The solution here is obvious but it has wide-ranging repercussions.

*Solution VI. Ensure general benefits (e.g. mortality/morbidity) exceed the general costs before recommending a public health or clinical application of a study showing a specific benefit*.

#### Examples

The benefits and costs of HRT in relation to post-menopausal symptoms (beneficial), and cardiovascular disease and cancer (not beneficial) are well known and were discussed earlier. Vitamin A at birth reduces mortality, at least in many Asian populations. However, it does not seem to produce the same benefits in Guinea-Bissau in Africa [35-37]. Even in India and Pakistan, the benefits are contested now, and it will not be beneficial in that setting for ever [35-37]. A recent study reported that reducing glycated haemoglobin to 6% in the elderly will improve control of diabetes, much desired by diabetes specialists, but increase mortality, an unfortunate and unexpected side-effect [38,39]. By contrast, Gaede *et al *reported a multifactorial intervention for people with diabetes that reduced non-fatal cardiovascular disease, progression to end-stage renal failure, and all cause mortality - perhaps an exemplary set of outcomes [40].

Following this solution would require larger trials, but in return the results would be more readily applicable in population settings. Where a trial can only demonstrate cause-specific benefits, the authors need to temper their public health-related conclusions, and consider the possibility of harmful effects that negate the benefits. Further, the net benefit of an intervention will be dependent on the mix of conditions in each population. To take a simple example, if an intervention reduces CHD but increases cancer, it will probably have net benefits in populations where CHD is common and cancer rare, but probably net harm where the opposite applies. In reality, trials will often not be powered to demonstrate general health benefits (except when these are claimed for the intervention), so information on harm may need to come from alternative sources, such as health monitoring data. Public health recommendations should be tentative until such data are available.

### Mistake 7: Failure by investigators and local health systems to utilise study data correctly to benefit health - a need for a higher authority providing a unified voice

Interpreting data correctly is among the highest intellectual endeavours in science, perhaps on a par with generating worthwhile hypotheses. Making health care and public health recommendations from data extends this skill. People who combine these skills are rare. The first skill needs razor sharp thinking on the scientific aspects of data, the second, similar capacity in relation to politics, policy, leadership, management and clinical care and public health. We can fail to apply data in two main ways - misapplication arising from misinterpretation, and non-application because the information has been set aside, or not brought to attention, or ignored.

There is an ethical imperative to act where it is warranted. Academic epidemiologists are, however, under pressure to research and teach, not to serve, and service-based clinicians and public health staff are under pressure to deliver services. The two worlds have been parting ways for some time [41,42]. The solution here is organisational.

#### Solution VII

Epidemiology needs to provide partners who apply research, including politicians, doctors, and public health specialists, with a unified voice. Is it not time for a World Council in Epidemiology and Causality that provides authoritative statements on epidemiological evidence and makes recommendation on when and how epidemiological data on associations are ready for application? I return to this question in the conclusions.

#### Example

The need for a unified voice is shown by 'Causality: A Frank Statement to Cigarette Smokers', the 1954 advertisement in US newspapers by 14 tobacco companies and trade associations, recently reprinted in the Lancet [43]. Among the statements about the relationship between tobacco and lung cancer were these:

"... experiments on mice have given wide publicity to a theory ...".

"... eminent doctors and research scientists have publicly questioned the claimed significance of these experiments."

"Distinguished authorities point out: That there is no agreement among the authorities regarding what the cause is "

(and 5 more points are made in a similar vein).

There will always be dissenting voices, and controversy can be exploited easily unless there is an authoritative voice that is trusted and independent. Such a voice is required to help apply important causal evidence, even when there is national hesitancy. Witness, for example, the failure to apply the knowledge that infants should be placed on their backs to sleep, to halve the risk of sudden infant death syndrome. Many European countries failed to apply this, leading to the unnecessary deaths of thousands of infants in Europe alone [44].

## Conclusion

There are numerous guidelines, published over some decades, on how to undertake and publish epidemiological research [45,46], available at the EQUATOR website http://www.equator-network.org/index.aspx?o=1032; accessed 14/8/08. These enjoy interesting acronyms such as CONSORT, STROBE, PRISMA, etc. Journals provide detailed guidance to authors and referees. Books summarise these for students. So why do we not apply the guidance? I think that our human frailties, the innate limitations of our science, insufficient education and training, and pressures of time and resources combine, making it hard to avoid the kind of fundamental mistakes illustrated in this paper. If so, the lessons are that:

1. we need to pay attention to the development of ethical and rigorous epidemiologists of high integrity [5,47] with high-level conceptual, theoretical and technical skills;

2. we make the innate limitations of our discipline more explicit;

3. we reorganise our scientific endeavour to make a collective, focused and more unified approach possible.

The seventh solution asks us to create a unified voice by pooling our intellectual resources, and creating a new global authority, to which I have given a provisional title. A World Council on Epidemiology and Causality could, however, dampen innovation. Alternatively, it could hasten advances, and counter the onslaught of undigested associations that bewilder us and will be multiplying as computerised data mining, data linkage, genetic epidemiology, and grand-scale epidemiology on millions of study participants become commonplace. (The analogy here is to do for associations and causality what the Cochrane collaboration, and the UK National Institute for Clinical Excellence, is doing for effectiveness of interventions.) Epidemiologists guard their academic freedom zealously and are mistrustful, sceptical people, particularly in relation to institutions. Nonetheless, to counter criticisms about false findings [18], advance epidemiology, and properly engage the public, we need to try out such a Council, learn from the experience and find workable, collaborative, global solutions to the kinds of problems illustrated here.

## Competing interests

The author declares that they have no competing interests.

## Authors' contributions

The author is solely responsible for this manuscript from conceptualisation to writing.
